# Condensate State as Determinant of Amyloid Pathology in Neurodegeneration

**DOI:** 10.3390/biom16040560

**Published:** 2026-04-10

**Authors:** Lathan Lucas, Josephine C. Ferreon, Allan Chris M. Ferreon

**Affiliations:** Department of Biochemistry and Molecular Pharmacology, Baylor College of Medicine, Houston, TX 77030, USA

**Keywords:** neurodegeneration, biomolecular condensates, fibrillation, amyloids, Tau, α-synuclein, amyloid-β, TDP-43, FUS, hnRNPA1

## Abstract

Neurodegenerative diseases arise when normally functional aggregation-prone proteins transition into stable cross-β amyloid fibrils. Although these fibrils share a conserved architecture, the pathways that lead to fibrillation vary across proteins and cellular environments. Liquid–liquid phase separation is now recognized as a central organizer of intracellular biochemistry that modulates protein aggregation. Physiological condensation can buffer aggregation by maintaining macromolecular solubility and providing partner interactions that compete against pathological protein–protein interactions. However, condensates can transform and age into gel-like states that can favor the emergence of β-rich oligomers and solid-state fibrils. Across six disease-linked proteins that include Tau, α-synuclein, amyloid-β, TDP-43, FUS, and hnRNPA1, we compare how sequence-encoded interaction motifs, cellular cofactors, and interfacial microenvironments shape the balance between physiological condensates and pathological amyloids. Here, we highlight the unifying drivers of aggregation and intervention points that preserve native function while limiting toxic amyloid formation.

## 1. Introduction

Proteins at the center of neurodegenerative diseases (NDs) transition from physiological forms that enable cell function to pathological amyloid fibrillar states that are generally functionally inactive [1,2]. A unifying feature of pathological fibrils is a cross-β architecture achieved largely through early anti-parallel or mature parallel β-sheet stacks [3,4]. The route to this thermodynamically stable state varies across proteins and often requires different contributions from specific aggregation intermediates, interactions with supramolecular interfaces, and dependence on key cellular environments [5,6,7]. In recent years, liquid–liquid phase separation (LLPS), a process by which macromolecules demix into concentrated, membraneless condensates, has emerged as a unifying principle of functional cellular organization with direct implications for pathogenesis [8]. Condensates can concentrate specific macromolecules, modulate reaction rates, and form in response to cellular stress [9,10,11,12]. Under certain conditions, these condensates can “age”, forming gel- and solid-like states that provide paths to amyloid formation [13,14]. Here, we highlight and connect pathways that differentiate physiological protein assemblies and condensates from those that lead to pathological amyloid formation across six ND-linked proteins.

**Figure 1:**
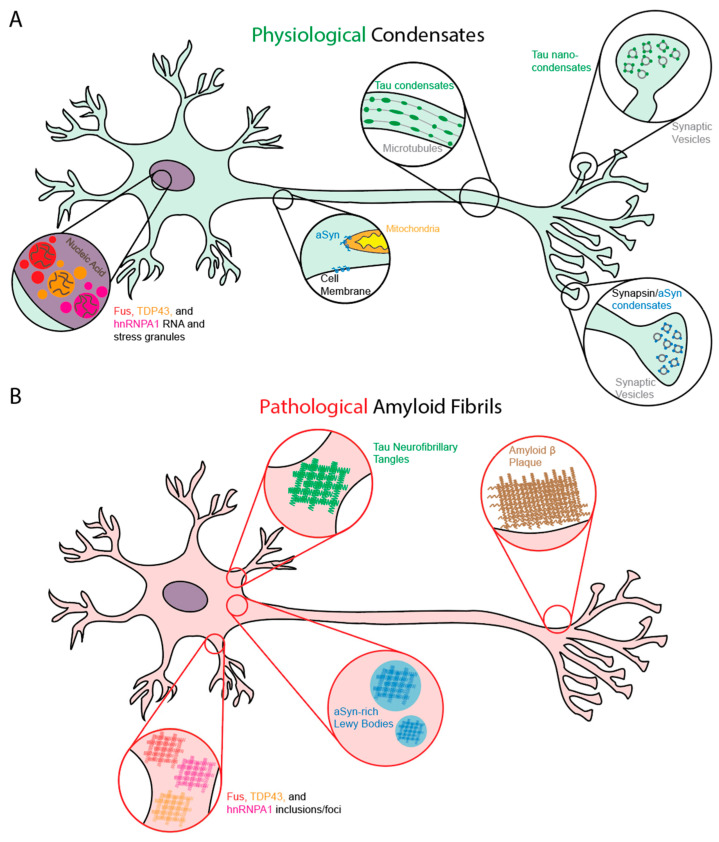
Functional vs. pathological landscapes of disease-linked proteins in neurons. (**A**) Physiological assemblies. FUS, TDP-43, and hnRNPA1 form dynamic nuclear RNA-rich condensates that regulate splicing and mRNA processing [8,9,15]. Tau forms condensates on axonal microtubules that support cytoskeletal organization and presynaptic nano-condensates that regulate synaptic vesicles [20,21,22,23]. α-Synuclein clusters on presynaptic vesicles and regulates vesicle cycling through mobile nanoscale condensates [25,26,27]. (**B**) Pathological amyloid states. In disease, these proteins convert to cross-β fibrils that are generally inactive [1,2]. Tau forms PHFs and SFs in NFTs [24]. α-Synuclein forms Lewy body and Lewy neurite fibrils [28]. Aβ accumulates as extracellular fibrils and plaques [29]. TDP-43, FUS, and hnRNPA1 appear in cytoplasmic amyloid inclusions with fibril cores derived from their LCDs [13,17,18,19].

Many ND-linked proteins can form both functional condensates in physiology and amyloid assemblies in disease. RNA-binding proteins (RBPs) like FUS, TDP-43, and hnRNPA1 form dynamic nuclear condensates with RNA that can coordinate various processes such as splicing, mRNA processing, and RNA export [8,9,15,16] (Figure 1A; Table 1). These same proteins can undergo condensate maturation toward β-sheet-rich, amyloid-like assemblies, with fibril-forming segments residing within their low-complexity domains (LCDs) [13,17,18]. TDP-43 forms cytoplasmic amyloid-like filaments in Amyotrophic Lateral Sclerosis (ALS) and Frontotemporal Lobar Degeneration with TDP-43 Pathology (FTLD-TDP) [19] (Figure 1B; Table 1). Likewise, Tau forms dynamic condensate-like assemblies on microtubules that locally concentrate Tau and tubulin to promote microtubule organization, and recent work has shown that Tau also forms presynaptic nano-biomolecular condensates that regulate the clustering and mobility of recycling synaptic vesicles [20,21,22,23] (Figure 1A; Table 1). In Alzheimer’s disease (AD) and other Tauopathies, Tau instead accumulates as paired helical filaments (PHFs) and neurofibrillary tangles (NFTs) [24] (Figure 1B; Table 1). Additionally, under physiological conditions, α-synuclein is enriched at presynaptic terminals where it binds/clusters synaptic vesicles and modulates SNARE-complex assembly; α-synuclein can also partition into synapsin:synaptic vesicle condensates while remaining highly mobile [25,26,27] (Figure 1A; Table 1). In Parkinson’s disease (PD) and Lewy body diseases, α-synuclein accumulates in Lewy bodies and Lewy neurites as abnormal filamentous assemblies [28] (Figure 1B; Table 1).

Amyloid-β (Aβ) differs from the other proteins discussed here in that it is not a native cytosolic protein, but rather a proteolytic fragment derived from the amyloid precursor protein (APP) [29,30]. Under physiological conditions, APP is primarily processed through the non-amyloidogenic pathway in which α-secretase cleavage occurs within the Aβ region of APP, followed by γ-secretase cleavage, thereby preventing generation of the Aβ peptide [29,31]. In contrast, the amyloidogenic pathway involves sequential cleavage of APP by β-secretase (BACE1) and γ-secretase, producing Aβ peptides of varying lengths, most commonly Aβ_1-40_ (consisting of 40 amino acids) and the more aggregation-prone Aβ_1-42_ (42 amino acids) [30,32]. To date, a defined physiological condensate of Aβ (40 or 42 amino acids in length) in neurons has not been established. Instead, reported Aβ condensation/LLPS has primarily been observed in reconstituted systems or on lipid membranes/cell surfaces, where it is discussed mainly in terms of modulating early amyloid nucleation and liquid-to-solid conversion rather than normal cellular organization [33,34,35,36] (Table 1). In any case, Aβ accumulates in the brain and deposits as β-sheet-rich amyloid in plaques, a core pathological hallmark of AD [29] (Figure 1B; Table 1).

These various proteins can exhibit recognized functional assemblies, but also transition into distinct, well-characterized amyloid forms in corresponding diseases. Current evidence indicates that LLPS can either accelerate or suppress amyloid formation depending on sequence-encoded interaction grammar, cofactors (e.g., RNA, small molecules), and condensate material state/aging [13,14,15,20,35,37,38]. Although this mechanistic framework has revealed multiple potential intervention points, translating these insights into therapies remains challenging because the same phase-separating interactions that promote pathological aggregation can also underpin essential physiological assemblies. Therefore, in this review, we perform a systematic cross-protein comparison of Tau, α-synuclein, Aβ, TDP-43, FUS, and hnRNPA1, linking experimentally validated fibril cores to LLPS sequence grammar, mapping fibrillation, and aligning these routes with physiological versus pathological LLPS. We reconcile contrasting findings across systems and identify potential intervention points that preserve native function while suppressing toxic assembly.

**Table 1:** Functional condensates/assemblies essential for neuronal function and pathological amyloids/aggregates prominent in various neurodegenerative diseases.

Protein	Functional Condensate/Assembly	Pathological Amyloid/Aggregate
FUS	Liquid nuclear condensates with RNA that support splicing and RNA processing [15].	Cytoplasmic amyloid inclusions in ALS and Frontotemporal Dementia (FTD) with fibril cores from the LCD [13].
TDP-43	Dynamic nuclear condensates that regulate RNA metabolism [39].	Cytoplasmic fibrils in ALS and FTD formed from ordered segments of the LCD [19].
hnRNPA1	RNA-rich nuclear condensates that support RNA processing [40].	Cytoplasmic fibrils in multisystem proteinopathy [41].
Tau	Condensates that organize microtubules within neuronal axons [21].	PHFs and NFTs in AD [24].
α-Synuclein	Mobile clusters on synaptic vesicles that regulate vesicle cycling and SNARE function [27].	β-sheet fibrils in Lewy bodies and Lewy neurites in PD [28].
Aβ	No confirmed physiological/functional condensate.	Extracellular fibrils and plaques in AD [29].

## 2. The Protein Sequence Grammar of LLPS and/or Fibrillation

### 2.1. Condensation-Driving Motifs

LLPS is a process in which macromolecules separate from the surrounding solution to form concentrated liquid-like condensates that lack a surrounding membrane [9]. In many biomolecular systems, LLPS occurs through electrostatic coacervation, in which oppositely charged macromolecules, such as intrinsically disordered protein:protein or protein:RNA pairs, associate through multivalent electrostatic interactions to form a dense liquid phase enriched in both components [9,13]. In other systems, LLPS can also be driven by hydrophobic and aromatic interactions, where multivalent contacts among nonpolar or π-interacting residues promote condensation of intrinsically disordered proteins into dense liquid phases [37]. Nonetheless, protein LLPS is encoded by sequence grammars that define multivalency and weak interactions among various “stickers” that are separated by flexible “spacers” [37,42,43,44,45]. Aromatic residues such as Y, F, and W and basic residues such as R (and in some contexts K) form π-π and cation-π contacts that act as stickers, whereas G- and S-rich segments and other polar spacer residues modulate chain solvation, conformational entropy, and effective valence [37,43,45]. Charge patterning, including the fraction of charged residues, net charge per residue, and blocky versus dispersed charge distributions, governs electrostatic interactions and single-chain conformations in disordered proteins that have been shown to tune electrostatic coacervation propensity and phase boundaries [46,47,48]. Recurrent LLPS-encoding features in RBPs include RGG repeats, SYGQ-rich low-complexity tracts, and enriched aromatic-basic patterning involving Y and R residues, as well as amphipathic helices and basic stretches that couple condensates to membranes or polyanionic partners [37,49,50,51]. Because LLPS behavior is encoded directly within intrinsically disordered regions, even modest sequence variation can shift phase boundaries and condensate material properties. Experimental studies have demonstrated that altering aromatic valence, charge patterning, or sticker distribution dramatically changes phase behavior in prion-like domains and other IDRs [37,42,43]. Consequently, differences between human and commonly used model organism protein sequences may produce distinct condensate behaviors or therapeutic responses, emphasizing the importance for researchers to validate LLPS mechanisms in human-derived systems.

**Figure 2:**
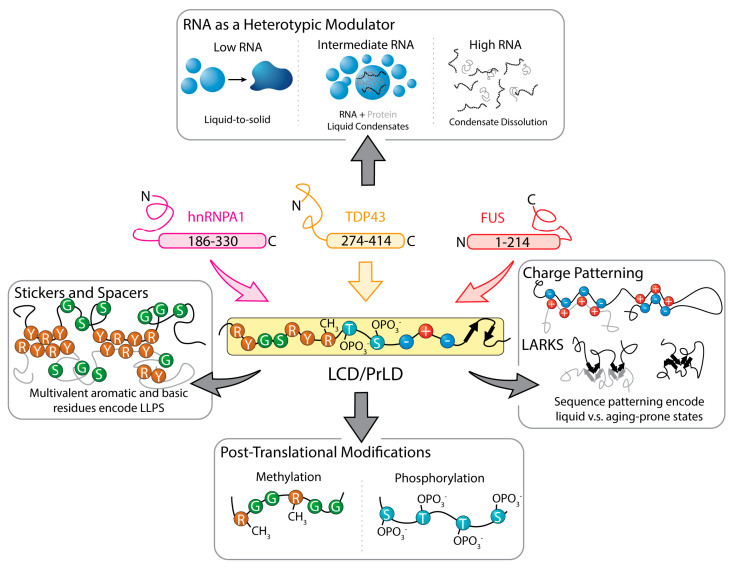
Sequence-encoded and context-dependent regulation of LLPS in RBPs. Representative RBPs (hnRNPA1, TDP-43, and FUS) contain LCDs or prion-like domains (PrLDs) that drive LLPS, despite differing domain positions across proteins. In hnRNPA1 and TDP-43, the LCD is C-terminal, whereas in FUS it is N-terminal. The central LCD schematic highlights intrinsic sequence features that encode LLPS, including multivalent aromatic and basic stickers separated by flexible G- and S-rich spacers, which tune solvation, conformational entropy, and effective valence [37,42,43,44,45]. Charge patterning, RGG repeats, SYGQ-rich tracts, and low-complexity aromatic-rich kinked segments (LARKS) further regulate phase boundaries and the propensity for liquid versus aging-prone condensate states [37,46,47,48,49,50,51,52,53]. Extrinsic factors modulate LCD-driven LLPS in a context-dependent manner. RNA acts as a heterotypic modulator of condensates, promoting LLPS at intermediate concentrations and dissolving condensates at high concentrations, yielding re-entrant phase behavior characteristic of ribonucleoprotein assemblies such as stress granules [11,15,37,54]. Post-translational modifications, including arginine methylation and phosphorylation, weaken sticker interactions and function as valence switches that suppress LLPS and pathological maturation [55,56,57].

For RBP LCDs, near-residue resolution mutagenesis has mapped an LLPS grammar in which Y and R residues provide the dominant sticker content and G- and S-rich residues function as spacers that tune solubility and saturation concentration (Figure 2) [37]. Related work on FUS-family prion-like domains shows that the valence and patterning of aromatic residues, particularly Y, play a central role in determining phase behavior [42]. These same LCDs, including those of FUS, hnRNPA1, and TDP-43, also contain low-complexity, aromatic-rich kinked segments (LARKS) that form kinked β-sheets and relatively weak cross-β networks, offering a structural mechanism by which liquid condensates may convert into more arrested gel-like states when dehydration or macromolecular crowding is increased (Figure 2) [52,53]. Thus, even at the level of LLPS grammars, the sequences of these RBP LCDs encode not only a propensity to condense but also a latent capacity to stiffen or age under specific environmental conditions.

RNA is a central heterotypic partner that tunes the phase behavior of the LCDs of RBPs in a strongly regime-dependent manner (Figure 2) [15,37]. In FUS, TDP-43, and hnRNPA1, RNA at intermediate concentrations provides multivalent anionic binding sites that enhance LLPS and maintain small, dynamic droplets, whereas RNA at higher concentrations saturates partner binding sites and dissolves droplets, modeled as a re-entrant phase diagram, in which increasing concentrations of the RNA binding partner first promote condensate formation but subsequently dissolve droplets once interaction sites become saturated (Figure 2) [15]. These liquid ribonucleoprotein (RNP) condensates form the core of stress granules, which reversibly sequester translation initiation factors to repress bulk translation during stress, and disassemble upon recovery to restore normal protein synthesis [11,54]. Post-translational modifications can also function as valence switches. R-methylation in RGG motifs weakens arginine-π contacts with aromatic residues, increases the saturation concentration, and suppresses LLPS of FUS-family LCDs (Figure 2) [55,56]. Phosphomimetic FUS LCD variants likewise disrupt LLPS and reduce aggregation and toxicity (Figure 2) [57].

**Figure 3:**
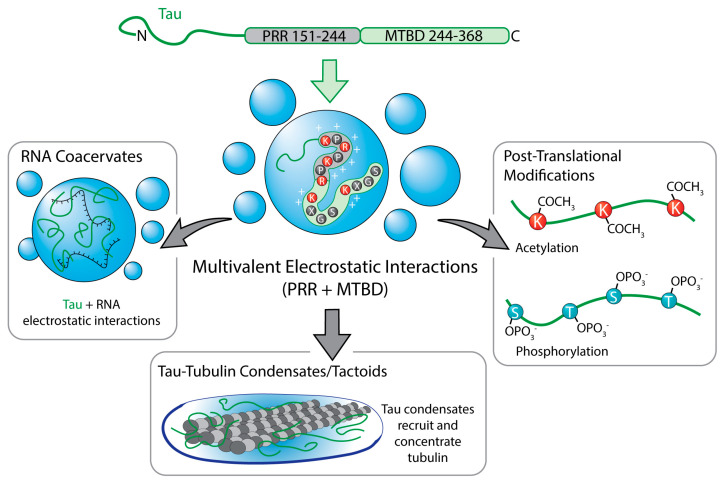
Electrostatic drivers of Tau liquid–liquid phase separation. Tau undergoes LLPS through multivalent electrostatic interactions distributed across multiple domains, rather than a single LCD. LLPS is driven primarily by positive charge interactions of the PRR (~151–244) and the MTBD (~244–368) with negatively charged N- and C-termini or other anions [58,59]. Phase behavior is strongly tuned by phosphorylation and phosphomimetic mutations [58,59,60,61,62,63]. In contrast, K acetylation suppresses Tau LLPS by neutralizing positive charge and inhibiting both spontaneous and polyanion-induced condensation [64]. Tau also forms electrostatically driven coacervates with RNA, yielding hydrated, reversible droplets with rapid internal dynamics [63]. A distinct functional regime emerges in Tau:tubulin condensates, where Tau recruits and concentrates tubulin to nucleate and bundle microtubules via heterotypic, largely electrostatic interactions [65,66].

Tau provides an example of how phase-separating interactions can be distributed across multiple modular regions rather than localized to an LCD. Tau LLPS has been shown to be driven primarily by multivalent electrostatic interactions involving the proline-rich region (PRR; ~151–244) and the microtubule-binding repeat domain (MTBD; ~244–368), where clusters of positively charged residues engage oppositely charged segments and cellular polyanions to promote condensation [58,59]. Within the MTBD, the classical PHF6* (VQIINK, 275–280) and PHF6 (VQIVYK, 306–311) hexapeptides define core amyloid-forming motifs that reside in this same LLPS-competent region [60]. While the MTBD is sufficient to undergo LLPS in vitro, particularly at 37 °C, in cultured mammalian cells, optogenetic mapping of Tau domains shows that the PRR is the dominant driver of condensate formation and that its phase behavior is strongly modulated by phosphorylation and phosphomimetic mutations (Figure 3) [61,62,63]. In contrast, K acetylation strongly suppresses Tau phase separation by neutralizing positive charge, thereby reducing multivalent electrostatic interactions [64] (Figure 3). This acetylation inhibits both spontaneous and polyanion-induced LLPS, and attenuates downstream aggregation pathways [64].

Tau:RNA complex coacervates represent a second LLPS grammar. Oppositely charged Tau and RNA form hydrated, reversible droplets in which Tau retains native-like conformations and rapid internal mobility, with electrostatics and sequence patterning setting the LLPS window (Figure 3) [63]. A distinct functional regime emerges when these grammars are engaged by tubulin. Tau forms liquid droplets capable of recruiting and concentrating tubulin [65,66]. Within these Tau:tubulin condensates, tubulin reaches locally high concentrations sufficient to nucleate and bundle microtubules, while Tau coats and scaffolds the resulting bundles (Figure 3) [65]. These reports are consistent with a heterotypic, largely electrostatic interaction between basic regions of Tau and acidic regions of tubulin driving this co-condensation, but a residue-level ‘grammar’ for Tau:tubulin LLPS has not yet been systematically mapped.

**Figure 4:**
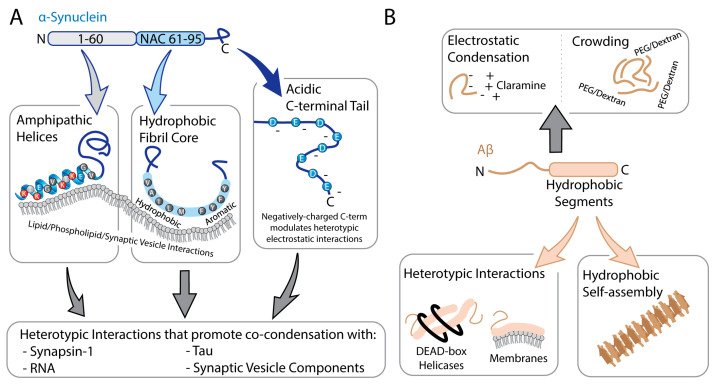
Drivers of LLPS for α-synuclein and Aβ. (**A**) α-Synuclein LLPS is driven by sequence elements that also mediate membrane binding and aggregation. The N-terminal amphipathic region (residues ~1–60) binds lipid surfaces, while the hydrophobic NAC region (residues ~61–95) forms the fibril core and contributes to condensate formation; the acidic C-terminal tail modulates condensation and maturation [67,68,69,70,71,72,73]. In cells, α-synuclein predominantly partitions into heterotypic condensates, including synapsin-1/synaptic vesicle assemblies and Tau:RNA droplets, where its recruitment and mobility are tuned by vesicle proteins and Tau post-translational modifications [27,75,76,77,78,79,80,81,82,83]. (**B**) Aβ undergoes LLPS primarily in heterotypic contexts, driven by electrostatic attraction to positively charged partners or scaffolds. Aβ_1-40_ forms condensates with cationic aminosterols such as claramine under crowded conditions, promoting liquid-to-solid transitions [35]. Hydrophobic segments in the central and C-terminal regions stabilize intermolecular contacts and drive self-association [84,85]. In condensates formed by DEAD-box protein LCDs, Aβ_1-42_ is sequestered and amyloid formation is suppressed, whereas crowding-induced or lipid-anchored condensates concentrate Aβ into nucleation-competent microdomains that accelerate fibril formation [33,36,86].

α-Synuclein provides a complementary example in which membrane-binding architecture and aggregation-prone segments intersect with LLPS behavior. The N-terminal region (~residues 1–60) adopts amphipathic helices on lipid surfaces, mediating high-affinity binding to micelles and vesicles (Figure 4A) [67,68,69]. The central non-amyloid-β component (NAC, residues ~61–95) is hydrophobic and forms the core of pathogenic fibrils, underscoring its aggregation-prone character [70,71]. Recent work on α-synuclein condensates indicates that the same N-terminal and NAC regions that engage membranes and form fibril cores also contribute to LLPS, while the acidic C-terminal tail modulates condensate formation and maturation (Figure 4A) [72,73]. In vitro, α-synuclein undergoes LLPS under macromolecular crowding or mildly acidic pH, conditions that enhance interactions involving the hydrophobic NAC region while reducing long-range electrostatic repulsion between molecules [72,74]. At synapses, α-synuclein is typically recruited into pre-existing condensates rather than forming large droplets independently. Synapsin-1, together with synaptic vesicles (SVs), creates the primary scaffold for these assemblies, and synapsin LLPS produces SV-rich droplets that subsequently recruit highly mobile α-synuclein. Within these condensates, α-synuclein remains largely diffused and is positioned to contribute to vesicle clustering and presynaptic function [27,75,76]. Recent work shows that VAMP2-containing SV mimetics can influence how α-synuclein partitions into vesicle-coupled condensates, indicating that specific vesicle proteins modulate α-synuclein recruitment to synapsin:SV assemblies (Figure 4A) [77]. Heterotypic assemblies of Tau and α-synuclein have also been observed in cellular and mouse models, where co-expression or inoculation leads to co-aggregation, cross-seeding, and mutual enhancement of pathology [69,70,71,72,73,74,75,76,77,78,79,80,81]. Consistent with these experimental observations, Tau and α-synuclein pathologies frequently co-occur in several human NDs, including Lewy body disorders that exhibit concomitant AD-type Tau pathology [82]. In vitro, Tau can form droplets through RNA-dependent phase separation, and full-length α-synuclein can readily partition into these Tau:RNA droplets through interactions between the α-synuclein C-terminal domain and Tau’s PRR. CDK2-mediated Tau phosphorylation enhances Tau enrichment in Tau:RNA droplets and suppresses α-synuclein incorporation, illustrating how post-translational modifications reshape the composition of these heterotypic condensates [83]. These studies support a model in which α-synuclein’s phase behavior is realized predominantly in heterotypic condensates.

Aβ can undergo LLPS in heterotypic contexts when paired with a positively charged partner, as shown for Aβ_1-40_ in the presence of the cationic aminosterol claramine under crowded conditions, where electrostatic attraction between the negatively charged peptide and the polyamine moiety of claramine drives condensate formation and promotes a liquid-to-solid transition (Figure 4B) [35]. Structural studies further show that hydrophobic segments in Aβ, including residues 16–21 and portions within 30–35, stabilize intermolecular contacts and promote self-association (Figure 4B) [84,85].

Condensates formed by LCDs from DEAD-box proteins, a family of RNA helicases implicated in Aβ regulation, sequester and locally concentrate the aggregation-prone Aβ_1-42_ peptide. Despite this increased concentration, heterotypic interactions with the LCD scaffolds inhibit amyloid fibril formation, rendering these biomolecular condensates protective reservoirs for Aβ_1-42_ (Figure 4B) [33]. By contrast, crowding-induced LLPS of Aβ in PEG/dextran polymer mixtures or lipid-driven Aβ condensates on supported bilayers generate condensed microdomains that act as nucleation sites and promote amyloid formation. In the lipid case, these condensates significantly accelerate the primary nucleation step and undergo liquid-to-solid transitions (Figure 4B) [36,86]. Thus, for Aβ, the hydrophobic interactions that drive its aggregation can either be buffered within condensates that sequester Aβ_1-42_ and suppress fibril formation, as seen for DEAD-box LCD-based coacervates, or concentrated into nucleation-competent microdomains, as seen for Aβ in PEG/dextran-induced droplets and lipid-anchored condensates on bilayers [33,36,86].

The proteins discussed above illustrate that LLPS grammars are not realized solely on intrinsic sequence properties, but are shaped by interactions with heterotypic partners such as RNA, cytoskeletal and synaptic scaffolds, membranes, and small molecules. More broadly, theoretical and experimental work shows that although motifs encoding LLPS specify a range of possible condensates, whether a given motif supports a functional, dynamic assembly or a condensate primed for pathological aging depends on stoichiometry, cofactors, post-translational modifications, and cellular context. This strong dependence on cofactors and cellular context also represents a major translational challenge, as condensate behavior observed in simplified in vitro systems may not directly reflect the complex molecular environments present in neurons.

### 2.2. Amyloid-Driving Motifs

Amyloid fibrils are stabilized by the canonical cross-β architecture in which β-strands run roughly perpendicular to the fibril axis and hydrogen-bond into β-sheets that stack along the axis [3,4,87]. At atomic resolution, many disease-associated fibrils segregate into steric-zipper spines, where two facing β-sheets interdigitate tightly enough to exclude water and form highly complementary “dry” interfaces [3,87,88]. Side-chain chemistry reinforces this packing in which hydrophobic and aromatic residues support Van der Waals and π-stacking interactions, Q and N form polar ladders through stacked hydrogen bonds, and salt bridges together with occasional cation-π contacts further stabilize and help specify particular sheet-to-sheet pairings [87,89,90]. In such structures, β-prone segments align to form extended β-sheets, and intramolecular turns link successive strands within each chain, building up the β-sheet layers that constitute the protofilament [4,91,92]. These structural principles help explain why short β-prone segments embedded in larger, often disordered proteins define fibril cores and exert strong influence over nucleation and the architecture of mature filaments [4,93].

**Figure 5:**
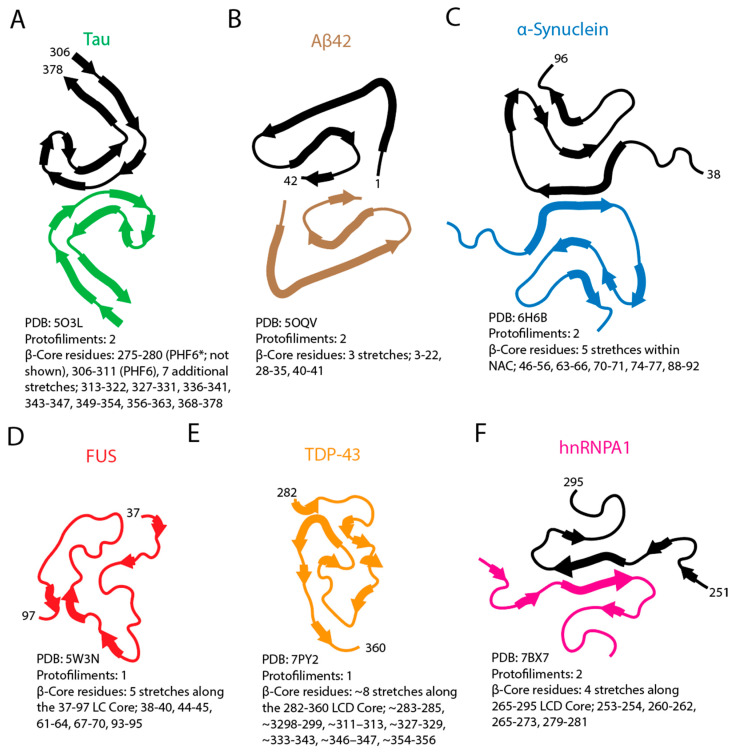
Side-by-side comparison of amyloid protofilament backbones from cryo-EM/ssNMR structural data. Backbone Cα traces of a single protofilament per structure, PCA-aligned and scaled for shape comparison: (**A**) Tau PHF protofilament (5O3L), (**B**) Aβ_1-42_ (5OQV), (**C**) α-synuclein (6H6B) NAC and flanking regions, (**D**) FUS (5W3N) LCD cores, (**E**) TDP-43 (7PY2) LCD, and (**F**) hnRNPA1 (7BX7) LCD core. All mature fibrils are typically parallel, cross-β, but differ in subunit shape, core length, and interface chemistry.

Patient-derived cryo-EM structures of Tau PHFs and straight filaments (SFs) from AD brains show that both polymorphs share a common ordered protofilament core comprising residues 306–378 within the MTBD, folding into a C-shaped cross-β/β-helix architecture. Two identical protofilaments assemble through distinct interfaces to give PHFs versus SFs (Figure 5A) [24,94,95]. Regions of Tau outside this ordered core remain structurally undefined in the cryo-EM reconstructions and correspond to disordered N- and C-terminal segments called the ‘fuzzy coat’ which project from the filament [24,60,94,96]. Within the AD filament core, the aggregation-prone hexapeptide PHF6 is incorporated into the ordered cross-β structure (Figure 5A), whereas the second hexapeptide PHF6*, although important for nucleation in vitro, is located outside the ordered core of AD Tau filaments [24,94,97].

Aβ fibrils resolved by cryo-EM from both in vitro Aβ_1-42_ preparations and human Aβ_1-42_ deposits in Alzheimer’s disease brains, like Tau, include polymorphs composed of two protofilaments (Figure 5B) [85,98]. In the Aβ_1-42_ fibril structure, the cross-β spine includes two prominent hydrophobic β-forming segments: the central KLVFFA motif (residues 16–21) and the C-terminal stretch encompassing residues 37–42, encoded by GGVVIA [85,99]. Both segments form dry-zipper interfaces that define the Aβ_1-42_ fibril. Importantly, multiple cryo-EM reconstructions from human AD brain show that while the exact protofilament interface varies, the β-forming segments themselves remain conserved (Figure 5B) [85,98,99].

α-Synuclein fibrils have well-defined amyloid grammar with their own structural logic. High-resolution structures from solid-state NMR of recombinant fibrils, cryo-EM of recombinant polymorphs, and cryo-EM of filaments from multiple system atrophy brain all reveal a compact cross-β core that spans much of the central hydrophobic region and strongly overlaps the NAC, while N- and C-terminal residues, similar to the Tau fuzzy coat, remain disordered and project from the core as flexible tails [70,100,101]. Biochemical and structural studies indicate that the central NAC region is critical for α-synuclein fibrillization and that deletions or disruptive point mutations within this hydrophobic stretch, including charge-introducing substitutions, can severely impair or abolish fibril formation [102,103,104]. The two common filament types observed for α-synuclein fibrils in multiple system atrophy differ in their protofilament folds and in how two distinct protofilaments pack together, although in both cases the β-strands are drawn from the same central sequence region (Figure 5C) [101,105]. Distinct α-synuclein filament conformations are associated with different synucleinopathies, with structures from multiple system atrophy differing from those observed in Lewy body diseases such as Parkinson’s disease and dementia with Lewy bodies [101,105]. Furthermore, in Lewy pathology, patient-derived α-synuclein filaments have an ordered cross-β core that fully encompasses the NAC region, consistent with extensive biochemical data showing that this hydrophobic segment is essential for fibril formation [102,105,106].

For the aggregation-prone ND-associated proteins discussed above (Tau, Aβ, and α-synuclein), metal ions are an important context-dependent class of modulators of amyloid formation in neurodegeneration. Aberrant levels of transition metals such as iron, copper, and zinc have been widely discussed in Alzheimer’s and Parkinson’s disease, where altered metal homeostasis correlates with amyloid plaque formation, Lewy body pathology, and oxidative stress in affected brain regions [107,108,109,110]. For Aβ, Cu^2+^ and Zn^2+^ bind the peptide and strongly influence aggregation behavior. Early studies showed that zinc can rapidly induce Aβ_1-40_ aggregation [111], and subsequent work has shown that Cu^2+^ and Zn^2+^ regulate Aβ assembly in a stoichiometry-dependent manner, sometimes favoring fibrils and in other settings stabilizing oligomeric or amorphous assemblies [112,113]. Similarly, transition metals can directly influence α-synuclein aggregation. Cu^2+^ and Fe^3+^ can alter α-synuclein oligomerization, fibril formation, and fibril morphology in vitro, but the direction and magnitude of these effects depend strongly on metal identity, stoichiometry, and protein variant [114,115,116]. Metal binding also influences Tau aggregation. Cu^2+^ binds directly to Tau monomer [117], Cu^2+^ and Zn^2+^ can promote Tau aggregation under some in vitro conditions [118], and iron dysregulation has been linked to increased Tau pathology in tauopathies [119,120,121]. Importantly, these metal-ion-linked amyloid aggregation studies were conducted largely without considering the proteins within phase-separated condensates, leaving open the question of how metal coordination might influence aggregation pathways within condensates where local protein concentrations, hydration states, and intermolecular interactions differ substantially.

In contrast to the dual-protofilament amyloid fibrils described above for Tau, Aβ, and α-synuclein, the RBPs FUS and TDP-43 contain LARKS that stack into kinked β-sheets and form weakly adhesive cross-β fibrils capable of building reversible hydrogel networks rather than tightly interlocked steric-zipper spines (Figure 5D,E) [53]. Regions with some overlap with the LARKS motifs can also participate in more stable amyloid-like cores as condensates age [122,123,124]. In FUS, ssNMR and biophysical analyses show that the LCD forms fibrils with a single cross-β core built from a ∼60-residue segment (residues 37–97) in which no β-strand exceeds six residues, yielding a non-extended backbone and a core that lacks the long hydrophobic β-strands typical of many pathogenic amyloids (Figure 5D) [52]. Earlier TEM and X-ray diffraction studies similarly showed that the FUS LCD assembles into amyloid-like fibers within hydrogels that exhibit a cross-β diffraction pattern, indicating an amyloid-like core in these gel-like polymers [125]. Disease-associated FUS mutations such as P525L accelerate the conversion of liquid FUS droplets into more solid, aggregated states [13].

TDP-43 behaves similarly to FUS with cryo-EM structures from ALS/FTLD patient tissue showing that the C-terminal LCD, particularly residues ~282–360, forms the ordered spine of the fibril with short β-strands and numerous turns, consistent with structural studies of aggregation-prone segments from the LCD (Figure 5E) [19,126]. Cryo-EM structures of pathological TDP-43 filaments extracted from ALS with FTLD brain demonstrate that residues 282–360 in the C-terminal LCD form the single protofilament ordered fibril core, which adopts a compact double-spiral fold composed of short β-strand segments arranged into a G-rich region (282–310), a hydrophobic region (311–342), and a Q/N-rich region (343–360) (Figure 5E) [19]. Cryo-EM studies of amyloid cores formed in vitro by isolated TDP-43 LCD segments similarly reveal short β-strand-rich folds, including the dagger-shaped conformers (residues 311–360) and the R-shaped fold (residues 286–331) [126].

hnRNPA1, another LCD-containing RBP, harbors short sequence segments that selectively promote amyloid formation. In the hnRNPA1 LCD, three motifs (SGSNFG, GSYNDF, and SSSSSY) are defined as fibril-forming segments (Figure 5F). Interestingly, the deletion of these segments strongly suppresses fibrillization while leaving LLPS largely intact [18,53]. Cryo-EM structures of amyloid fibrils formed in vitro from hnRNPA1 LCD and full-length hnRNPA1 show that both fibrils comprise two protofilaments related by an approximate twofold/pseudo-2_1_ screw symmetry (Figure 5F) [127,128]. In the LCD fibril, the protofilaments contact through a small, well-defined interface built around a polar steric zipper, with flanking F and other hydrophobic side chains forming π-stacking and hydrophobic contacts that tighten protofilament packing [127].

## 3. The Multi-Faceted Relationship Between Condensation and Fibrillation

### 3.1. Liquid Condensates Can Resist Amyloid Fibrillation

In several systems, physiological condensates reduce susceptibility to amyloid formation by remaining liquid and favoring functional homo- and hetero-typic contacts over self-templating β-interactions. For FUS, TDP-43, and hnRNPA1, heterotypic partners and RNA-rich environments can hold these proteins in dynamic condensates and reduce their progression to irreversible amyloid. For TDP-43, binding to poly(ADP-ribose) (PAR) promotes LLPS and recruitment into stress granules, where short-term stress-granule localization initially protects TDP-43 from disease-associated phosphorylation and granulo-filamentous aggregation, even though prolonged stress eventually leaves behind aggregates of phosphorylated TDP-43 [129].

RNA binding and recruitment of cytoplasmic FUS into stress granules protects the protein from irreversible aggregation, indicating that RNA-rich, stress-granule-like condensates can act as transient safe harbors for RBPs [130]. hnRNPA1 condensates can be rendered strongly aggregation-resistant without abolishing LLPS. The hnRNPA1 LCD forms droplets whose interfaces normally promote fibril nucleation, but coating that interface with small-molecule or protein surfactants preserves LLPS while delaying or arresting Thioflavin-T (ThT)-positive fibrils and starburst-like assemblies [18]. Complementary work on full-length hnRNPA1 shows that RNA:protein stoichiometry redirects both phase behavior and aggregation pathways. At low RNA levels, RNA promotes condensation and accelerates ThT-positive amyloid formation (with fibrils confirmed by EM), whereas at higher RNA:protein ratios condensation is suppressed by re-entrant behavior yet amyloid formation can still emerge over longer timescales [15,131].

Loss of TDP-43 function has been shown to deplete long pre-mRNAs and induce missplicing in neuronal transcripts, supporting a broader model in which TDP-43 and FUS participate in RNA processing [132,133]. At the level of condensate material state, TDP-43 function has been probed directly with splicing reporters. An N-terminal mutation that disrupts NTD polymerization weakens LLPS, increases droplet fluidity, and reduces splicing activity [134]. In contrast, ALS-associated mutations in the CTD helical region disrupt helix-mediated self-interaction and generally reduce phase separation, suggesting a potential loss of normal TDP-43 assembly and function [135]. For FUS, nuclear import receptors such as Kapβ2 inhibit and reverse aberrant phase transitions by dispersing liquid droplets, hydrogels, and pre-formed fibrils, thereby suppressing oligomerization and fibrillar assembly [136,137]. In parallel, dynamic RNA binding to FUS maintains highly mobile condensates and prevents pathological phase separation, whereas loss of RNA interaction promotes aberrant LLPS and aggregation [138].

Tau exhibits LLPS behaviors that remain aggregation-neutral or resist amyloid conversion. Tau undergoes LLPS with RNA to form electrostatically driven, hydrated complex coacervate droplets that are reversible and dynamically liquid under near-physiological salt and temperature conditions [139,140]. In these Tau:RNA droplets, Tau retains solution-like conformational dynamics and hydration despite high local concentration, as shown by EPR/DEER and droplet fusion assays [139]. Under the equilibrium LLPS conditions, Tau:RNA condensates remain dynamic and non-fibrillar over many hours, suggesting that Tau can occupy a metastable liquid state that does not obligatorily undergo amyloid conversion [140]. Tau repeat domain (RD)-based cellular reporter systems, including CFP/YFP FRET biosensors and RD-YFP reporter lines, provide highly sensitive readouts of templated Tau aggregation in living cells [141,142,143]. Using the FRET-based biosensor line, phase-separated and aged Tau droplets have been shown to generate robust FRET-positive inclusions, whereas soluble Tau produces little or no signal, providing a functional readout of pathogenic conversion [20]. In parallel, in vitro studies of hydrophobically driven, high-salt Tau droplets reveal a tight coupling between LLPS and fibrillization, whereas electrostatically driven Tau:RNA coacervates remain reversible and largely non-fibrillar, supporting a model in which relatively short-lived, electrostatically driven physiological condensates are largely non-seeding, whereas aged or hydrophobically driven droplets become strongly amyloidogenic [20,143]. Tau also forms liquid condensates along microtubules that enrich tubulin and promote microtubule bundling. Within these compartments, Tau remains diffusible and liquid-like around the bundles, consistent with a dynamic microtubule-scaffolding role [65]. Tau-microtubule functionality has been quantified using microtubule polymerization assays and TIRF imaging of microtubule growth within Tau condensates [65], together with classic Tau-tubulin binding and assembly assays, including polymerization and co-sedimentation-type readouts [144,145]. Consistent with this, tubulin partitions into Tau:α-synuclein condensates to maintain Tau and α-synuclein in physiological, microtubule-engaged condensates and suppress their oligomerization and aggregation, showing that a heterotypic partner can block pathological maturation [66].

Several α-synuclein systems also demonstrate LLPS-mediated protection. β-Synuclein partitions into α-synuclein condensates, where it slows liquid-to-solid maturation and reduces amyloid conversion, as demonstrated by fluorescence recovery after photobleaching (FRAP), ThT, and solid-state NMR measurements of β-structure in co-condensates [146]. Other studies show that β-synuclein:α-synuclein condensates limit droplet fusion and growth, thereby disrupting α-synuclein condensate maturation [147]. Condensates formed by non-aggregating components such as ATP or RNA can either accelerate aggregation when α-synuclein accumulates at their interface or sequester and stabilize α-synuclein to suppress aggregation [148]. At synapses, synapsin-mediated vesicle condensates physiologically recruit highly mobile α-synuclein, where it remains dynamically partitioned within liquid-like assemblies [27]. Synapsin-mediated condensates have been probed using reconstituted synaptic-vesicle clustering assays and live imaging of vesicle recruitment and mobility in the presence of α-synuclein [27], providing a functional counterpoint to the low-mobility condensates that mature into amyloid-rich hydrogels during pathological α-synuclein condensation [149].

Aβ_1-42_ has a high tendency to aggregate into soluble oligomers and fibrils, and accumulation of Aβ_1-42_ and related Aβ species is strongly implicated as an early, often initiating driver of Alzheimer’s disease [29,150]. Despite its strong intrinsic aggregation propensity, Aβ_1-42_ can be sequestered into biomolecular condensates formed by low-complexity DEAD-box protein domains, where high local peptide concentration does not lead to fibrillization. Confocal imaging and partition analysis show that Aβ_1-42_ is highly enriched within these condensates, while ThT aggregation kinetics, size-exclusion chromatography, and TEM reveal that fibril formation is strongly delayed or fully suppressed and a substantial fraction of Aβ_1-42_ remains monomeric. Together, these data demonstrate that certain condensate environments can act as protective ‘safe reservoirs’ that buffer Aβ_1-42_ against amyloid conversion rather than promoting it [33].

The examples described above show that when condensates remain liquid and properly buffered by RNA or heterotypic partners, they reduce β-competent homotypic contacts and divert aggregation-prone proteins into non-pathogenic interaction networks. This supports therapeutic strategies that modulate condensate composition and interfaces rather than indiscriminately disrupting LLPS.

### 3.2. Liquid Condensate Environments Can Promote Amyloid Fibrillation

Condensates can promote amyloid formation by creating locally supersaturated environments that enhance nucleation [8], consistent with classical nucleation theory in which increasing supersaturation lowers the free-energy barrier for phase conversion [151]. Condensate organization also creates microenvironments that favor fibril nucleation. Macromolecular enrichment and multivalent interaction networks increase molecular encounter frequencies and stabilize otherwise rare interaction states, thereby raising the probability of amyloid nucleation [9,50]. The altered physicochemical environment inside condensates, including high local macromolecular concentration and enriched multivalent interactions such as π-π, cation-π, and hydrophobic contacts, is expected to favor aggregation-prone states and increase the risk of amyloid [50].

**Figure 6:**
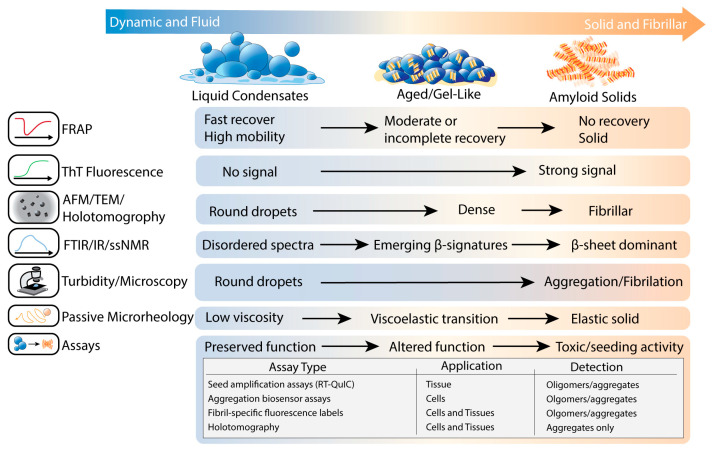
Schematic illustrating how commonly used biophysical, structural, and functional assays interrogate distinct material states along the condensate-amyloid continuum. Condensates transition from dynamic liquid droplets to aged or gel-like assemblies and ultimately to solid amyloid aggregates. Techniques positioned beneath each regime report complementary properties, including molecular mobility (FRAP), phase behavior and morphology (turbidity and microscopy), viscoelasticity (passive microrheology), secondary structure formation (ThT fluorescence, FTIR/IR), ultrastructure (AFM and TEM), and functional or seeding activity (seed amplification, FRET-based biosensors, fibril-specific fluorescent labels, holotomography).

These liquid-to-solid transitions have been captured across multiple biophysical techniques. FRAP reveals the progressive loss of molecular mobility during condensate aging [13], and passive microrheology quantifies the corresponding increase in viscoelasticity and emergence of gel-like behavior (Figure 6) [152]. Emerging β-structure can be monitored through ThT fluorescence and infrared spectroscopy under aggregation-permissive LLPS conditions (Figure 6) [153], while electron and atomic-force microscopy directly visualize fibrils at or near condensate interfaces when they appear (Figure 6) [18].

As condensates age, disease-linked mutations and/or cellular stressors can drive an increase in interaction lifetimes and a decline in internal mobility. These molecular changes can convert initially liquid droplets into viscoelastic or gel-like states that more readily support fibril formation [13,14,152]. FUS droplets exemplify this transition. ALS-linked mutations and FTLD-linked hypomethylation accelerate the maturation of FUS condensates from RNA-containing condensates into solid-like assemblies [13,55]. TDP-43 condensates also undergo stress- and mutation-dependent maturation. Under oxidative stress, TDP-43 is recruited into RNA-rich stress granules, and prolonged or chronic stress conditions give rise to cytoplasmic phospho-TDP-43 aggregates that coexist with or outlast stress granules in patient-derived fibroblasts and iPSC-motoneurons [129,154]. ALS-linked mutations that weaken N-terminal oligomerization or disrupt the C-terminal helical interaction region reduce TDP-43 phase separation and self-interaction, and a single N-terminal phosphomimetic mutation similarly suppresses LLPS and impairs RNA splicing in minigene assays [134,135]. The hnRNPA1 LCD shows a similar behavior. Under standard buffer conditions in the absence of RNA, the hnRNPA1 LCD phase separates into droplets that spontaneously nucleate amyloid fibrils at their interfaces, with ThT fluorescence, confocal imaging of ThT-positive rims, and transmission electron microscopy/atomic force microscopy (TEM/AFM) visualization of starburst fibrils marking an overall liquid-to-solid transition of the condensate phase (Figure 6). Deleting short amyloid-promoting segments or coating the droplet surface with small-molecule or protein surfactants preserves LLPS, as evidenced by sustained droplet formation in bright-field microscopy, but strongly reduces ThT signal and interface-localized fibrils in EM/AFM, indicating that sequence grammar and interfacial chemistry, rather than phase separation alone, control amyloid output from hnRNPA1 condensates [18].

Distinct LLPS regimes in Tau highlight how condensate type and aging together shape aggregation risk. Electrostatic Tau:RNA complex coacervates form within a narrow, stoichiometrically balanced window where droplets remain hydrated, reversible, and dynamic for many hours, with ThT fluorescence and electron microscopy showing no detectable fibril formation under these conditions [139,140]. In contrast, high-salt-induced Tau condensates rely on electrostatic screening and enhanced hydrophobic contacts, and in this regime, ThT aggregation kinetics, increased dehydration measured by Electron Spin Echo Envelope Modulation and Overhauser Nuclear Dynamic Polarization, and EM all show that β-structure accumulation and fibrillization are tightly coupled to condensate aging [143]. Independent work studying crowding-induced Tau condensation in near-physiological buffers shows a similar aging trajectory, where FRAP measurements reveal that initially liquid droplets become gel-like within minutes and, over days, Thioflavin S staining and a Tau RD-P301S FRET-based biosensor assay demonstrate that these aged condensates contain Tau aggregates capable of seeding cellular Tau aggregation (Figure 6) [20]. Phosphorylation in the Tau PRR has been shown to promote phase separation in cells and in vitro [62,155], and more global phosphorylation patterns that include PRD sites enhance Tau phase separation into gel-like condensates and accelerate formation of AD-like amyloid filaments over time [156]. Heterotypic protein condensates such as Tau:prion complex coacervates, which can be tuned by RNA into multiphasic droplets, gradually age into solid-like co-assemblies containing amorphous and amyloid-like aggregates [157]. Separately, small molecules and ions have been shown to accelerate or delay the liquid-to-solid transition of phase-separated Tau condensates, demonstrating that Tau droplet aging kinetics are chemically tunable and suggesting a route to modulate pathological maturation [158].

It is worth noting that not all arrested or gel-like condensates are on an inevitable path to fibrillation. In Tau condensates, the aggregation inhibitor methylene blue promotes LLPS and accelerates Tau liquid-to-gel condensate maturation, yet it inhibits conversion of droplets into amyloid fibrils and reduces aggregate cytotoxicity [159]. Methylene blue’s reduced derivative leucomethylthioninium (LMTM) also suppresses Tau fibrillization, as demonstrated in biochemical assays and PHF dissolution studies, also contributing to liquid-to-gel transition of Tau condensates [160,161,162,163]. Similar behavior has been reported for polyphenols such as gallic acid and tannic acid. Gallic acid acts as a concentration-dependent biphasic modulator of Tau LLPS that accelerates the liquid-to-gel transition of Tau condensates while effectively impeding the formation of deleterious fibrillar aggregates [164]. Tannic acid similarly modulates Tau LLPS in a biphasic manner, promoting phase separation at low tannic acid:Tau ratios and suppressing it at higher ratios, and together with biochemical evidence, these findings support the idea that such polyphenols can tune Tau condensate material properties and, under appropriate conditions, help limit conversion of Tau droplets into fibrillar aggregates [165,166]. Across these systems, protective Tau gels were identified by combining LLPS and material-state readouts. Turbidity and droplet imaging together with FRAP or droplet relaxation assays to document liquid-to-gel transitions were combined with aggregation assays such as ThT fluorescence, EM, and cell-viability measurements to show that, despite slowed internal dynamics, these condensates greatly delay or suppress the emergence of ThT-positive, EM-visible fibrils and associated cytotoxicity (Figure 6) [159,164,165].

In α-synuclein, condensate environments and aging often promote aggregation. In vitro, recombinant α-synuclein forms liquid-like droplets that gradually lose internal mobility as measured by FRAP, undergo a liquid-to-gel transition, and ultimately convert into amyloid-rich hydrogels, as shown by ThT fluorescence and electron microscopy (Figure 6) [148]. Conceptually, α-synuclein LLPS is proposed to promote aggregate formation that interferes with PINK1-PRKN-mediated mitophagy, linking condensate-driven α-synuclein aggregation to mitochondrial quality-control defects in PD models [167,168]. LLPS can also modulate fibril polymorphism, with fibrils grown from aged α-synuclein droplets displaying greater structural heterogeneity detectible by AFM and Fourier-transform infrared spectroscopy (FTIR) than those formed in dilute solution (Figure 6) [169]. Certain α-synuclein co-condensates provide an additional LLPS-dependent route to aggregation. Ubiquilin-2 undergoes LLPS to form liquid droplets that recruit α-synuclein. As these Ubiquilin-2 droplets transition from a liquid to a gel/solid-like state, the α-synuclein sequestered within them converts into pathogenic fibrils both in vitro and in cells [170]. In cells, α-synuclein was driven into liquid condensates using a multivalent α-synuclein-emGFP-5Fm scaffold, which enabled controlled assembly of liquid droplets whose aging state was verified by FRAP (Figure 6) [171]. Upon addition of exogenous α-synuclein fibril seeds, these condensates underwent a liquid-to-solid transition and generated amyloid-positive structures, as demonstrated by FRAP, amyloid-binding dyes, and pathological pS129 species immunostaining (Figure 6) [171].

Aβ also shows condensate-driven aggregation in specific crowded regimes. Under PEG/dextran crowding conditions, Aβ undergoes LLPS into droplets whose interfaces act as heterogeneous nucleation sites for fibril formation, where varying electrolyte identity modulates both droplet formation and the kinetics of Aβ fibrillation [86]. Additionally, Aβ also forms membrane-anchored condensates through lipid-driven LLPS in vitro and in living cells. In these systems, Aβ_1-42_ assembles into liquid droplets that remain tightly coupled to the bilayer surface and then mature into solid-like assemblies that accelerate the primary nucleation step in the amyloid conversion cascade. These transitions were defined using total internal reflection fluorescence (TIRF) and confocal imaging to visualize membrane-bound condensates and FRAP to show initially liquid internal dynamics that slow with aging (Figure 6) [36]. Complementary studies show that soluble Aβ oligomers and Aβ_1-40_ can also undergo LLPS in surfactant-containing or crowded solutions, where liquid droplets provide an intermediate state that accelerates amyloid fibril formation [35,86,172]. Once a fibril nucleus is present within or near a condensate, local enrichment of proteins near the nucleus would be expected to favor rapid elongation and fibril-surface secondary nucleation. In Aβ_1-42_ systems, quantitative kinetics have shown that after a small seed population forms, toxic oligomers arise predominantly through fibril-catalyzed secondary nucleation rather than primary nucleation in solution [173].

**Figure 7:**
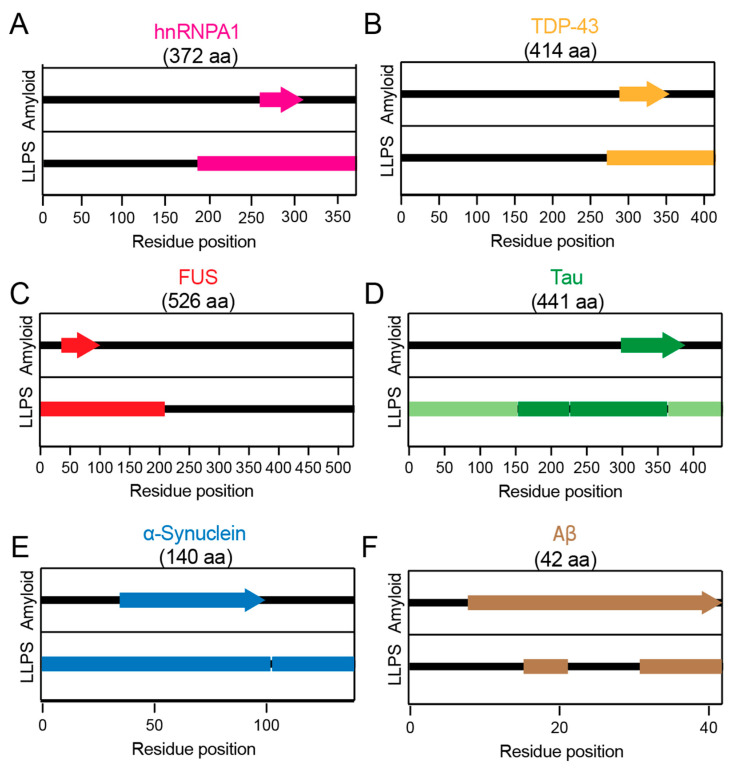
Maps of LLPS- and amyloid-driving regions across major neurodegeneration-associated proteins. Schematic representations summarize the approximate sequence locations that have been experimentally implicated in LLPS versus amyloid fibril formation for each protein. Black bars represent the full-length protein sequence, colored segments denote regions with dominant LLPS- or amyloid-promoting behavior, and arrows indicate regions that nucleate or propagate fibrillar assembly. (**A**) hnRNPA1, the C-terminal LCD (~residues 186–372) drives LLPS [18,40], while overlapping segments within the distal C-terminus preferentially support amyloid formation upon condensate maturation or disease-linked perturbations (PDB: 7BX7). (**B**) TDP-43, LLPS is primarily mediated by the C-terminal LCD (~residues 274–414) [39], which also harbors short amyloid-prone motifs that nucleate fibril formation in pathological states [19] (PDB: 7PY2). (**C**) FUS, The N-terminal prion-like LCD (~residues 1–214) robustly drives LLPS [13,15], whereas discrete N-terminal segments within the FUS low-complexity domain can form ordered cross-β fibrillar cores, while the remainder of the domain stays dynamically disordered [52] (PDB: 5W3N). (**D**) Tau, LLPS is primarily promoted by multivalent interactions between the positively charged PRR and MTBD (~residues 150–360, shown in dark green) with the negatively charged N- and C termini (shown in light green) [58,59,61,62,63]. Amyloid formation arises from β-competent motifs within the MTBD and adjacent C-terminal regions that become exposed or stabilized during condensate aging (PDB: 5O3L). (**E**) α-Synuclein, LLPS involves much of the intrinsically disordered sequence, with contributions from both the N-terminal amphipathic region and the acidic C-terminus [74,149,174]. Amyloid fibril formation is driven predominantly by the central NAC and its flanking regions (~residues 46–69, PDB: 6H6B). (**F**) Aβ, short hydrophobic segments drive both weak phase separation and rapid amyloid assembly, particularly with respect to lipid-bound condensation [34,36,84,85], with the central hydrophobic cluster and C-terminal residues serving as the principal fibril-forming elements [84,85] (PDB: 5OQV).

Across neurodegeneration-associated intrinsically disordered proteins, regions that promote LLPS frequently overlap with those that drive amyloid assembly. As summarized in Figure 7, these overlapping sequence segments, often LCDs or PrLDs enriched in multivalent interaction motifs, support dynamic condensation under physiological conditions but can also nucleate β-sheet-rich fibrils upon condensate aging, mutation, or environmental stress. Mapping these regions highlights a shared molecular grammar underlying functional LLPS and pathological aggregation, emphasizing that disease emerges not from distinct sequences, but from altered regulation of the same interaction-prone domains.

## 4. Therapeutic Considerations for Targeting Pathological Condensate Regimes

An obstacle for therapeutic development is that condensates exist along a continuum of material states ranging from highly dynamic liquids to gel-like intermediates and solid amyloid assemblies, complicating efforts to determine which stage of the transition represents the most tractable and selective therapeutic target. Broad suppression of LLPS is unlikely to provide therapeutic benefit, as indiscriminate condensate dissolution disrupts both physiological and pathological assemblies. For example, 1,6-hexanediol dissolves condensates by weakening hydrophobic interactions but lacks regime specificity and remains primarily a biophysical probe rather than a therapeutic agent [175,176,177,178] (Table 2). Similarly, perturbing RNA buffering in prion-like RBPs can shift systems into more persistent and aggregation-prone regimes rather than restoring healthy dynamics [15] (Table 2).

Strategies focused solely on clearing late fibrillar species also show limited efficacy. Clinical anti-Aβ antibodies such as lecanemab and donanemab reduce plaque burden, but yield modest clinical benefit, consistent with the possibility that upstream oligomeric or condensate-related pathogenic regimes remain incompletely addressed [179,180,181,182] (Table 2). Tau-directed small molecules such as hydromethylthionine and methylene blue further illustrate this principle, as they redirect Tau into gel-like condensate states that suppress fibril formation without abolishing assembly, yet still face challenges in clinical translation [159,183,184,185] (Table 2). These outcomes indicate that suppressing end-stage aggregates without reshaping the regimes that generate toxic species is unlikely to be sufficient. More broadly, dysregulated condensation has recently been proposed as a unifying disease mechanism termed “condensatopathies,” encompassing conditions in which aberrant condensate formation, composition, or material state drives pathology [186]. Within this framework, pathological condensates are viewed not only as mechanistic intermediates in disease progression but also as potential diagnostic and therapeutic targets.

Multiple classes of agents selectively target pathological condensate regimes while preserving or restoring physiological ones. Import receptors such as Kapβ2 reliquefy aberrant FUS assemblies and restore nuclear localization, while small molecules including lipoamide, lipoic acid, and selected RNA-interacting scaffolds soften pathological stress granule states without globally blocking stress responses [136,137,138,187,188] (Table 2). Related compounds such as α-lipoic acid have entered clinical investigation for NDs including ALS, illustrating how modulation of condensate behavior may translate to therapeutic development [189,190]. Additional compounds such as bis-ANS, TMAO, and planar aromatic RNA-binding molecules demonstrate that liquid, intermediate, and solid regimes can be differentially tuned in a concentration- and context-dependent manner [135,191,192,193] (Table 2). Some RNA-binding molecules, such as mitoxantrone, are DNA-intercalating and are clinically used anticancer and immunomodulatory drugs [194]. Although shown to disrupt RNA-mediated condensate assembly and reduce recruitment of aggregation-prone RBPs into stress-granule condensates [195], mitoxantrone’s cellular toxicity and limited efficacy have restricted their application in neurodegeneration [193,196,197,198].

Parallel regime-selective effects are observed for Tau and α-synuclein. Small molecules, including methylene blue, polyphenols, suramin, and claramine, stabilize non-fibrillizing liquid or gel-like condensates, suppress nucleation within droplets, and reduce downstream toxicity [38,159,164,165,199,200,201] (Table 2). These studies support a therapeutic framework in which pathogenic condensate trajectories are diverted or neutralized rather than globally suppressed.

An important goal in the field is to integrate diagnostic and therapeutic strategies that target the same molecular pathology, which to date has been largely focused on end-stage aggregates. In AD, positron-emission tomography (PET) tracers targeting Aβ and, more recently, Tau aggregates are now widely used to detect pathology in vivo and to guide patient selection and monitoring in anti-amyloid therapeutic trials [202,203,204,205,206]. Diagnostic seed amplification assays such as RT-QuIC, originally developed for prion diseases, have been adapted to detect α-synuclein aggregation seeds in cerebrospinal fluid from patients with synucleinopathies (Figure 6) [207,208]. Continued efforts to understand the molecular mechanisms that govern early aggregation events therefore remain essential for developing more effective interventions. Thus, as the structural and biochemical determinants of protein condensates and their maturation states become clearer, similar strategies may eventually enable detection and therapeutic targeting of condensate-mediated aggregation pathways.

Collectively, effective therapies will not abolish condensates but instead reshape their material states. Stabilizing protective liquid regimes, diverting aggregation-competent intermediates, and neutralizing arrested or seeded condensate states emerge as a more realistic strategy than targeting LLPS or fibrillation in isolation, and provides a coherent rationale for combination therapies that pair aggregate-directed agents with regime-shaping modulators (Table 2).

**Table 2:** Small molecules and compounds that, to date, have been tested to target various condensate regimes.

Compound or Agent	Primary Target	Condensate Regime Targeted	Mechanism
1,6-hexanediol	Broad protein condensates	Physiological and pathological LLPS	Weakens hydrophobic interactions indiscriminately, dissolving many condensates without regime specificity. Useful as a probe rather than a therapeutic [175,176,177,178].
Lecanemab	Aβ	Late fibrillar regime	Binds aggregated Aβ including protofibrils and promotes plaque clearance but does not directly address upstream oligomerization or condensate-driven nucleation [179]
Donanemab	Aβ	Late fibrillar regime	Reduces plaque burden with modest clinical benefit, suggesting limited impact on earlier pathogenic regimes [180]
Kapβ2	FUS and related RBPs	Pathological solid-like condensates	Binds NLS and LCD, reliquefies hardened condensates, suppresses fibril formation, and restores nuclear localization while preserving functional condensates [136,137,138,187]
Lipoamide	FUS and stress granule RBPs	Pathological stress granule regimes	Partitions into, prevents and dissolves stress granules, but does not significantly alter stress-impaired translation [188].
Lipoic acid	FUS and stress granule RBPs	Pathological stress granule regimes	Reduces stress granule reporter aggregation in vivo and improves ALS model motor phenotypes [188].
Bis-ANS	Broad protein condensates	Liquid versus solid transition regimes	Promotes liquid-like droplets at low concentrations but disrupts droplets at higher concentrations [191,209].
TMAO	TDP-43 LCD	Liquid condensates versus fibrils	Promotes liquid-like condensation while suppressing amyloid fibril formation, demonstrating separability of regimes [192].
Mitoxantrone	TDP-43 and FUS in stress granules	Pathological RNA-dependent condensates	Reduces RNA-dependent recruitment of ALS-linked RBPs into stress granules and reduces persistent cytoplasmic puncta [193,195].
RNA oligonucleotides	hnRNPA1	Pathological cytoplasmic condensates	Target defined RRM interfaces to reduce aggregation and improve downstream cellular functions [210,211,212].
Short RNAs	FUS and TDP-43	Pathological cytoplasmic condensates	Dissolve aberrant condensates and mitigate proteotoxicity by restoring RNA-mediated buffering [213,214].
ATP	Broad RBP systems	Physiological liquid regimes	Acts as a hydrotrope at physiological millimolar concentrations, maintaining protein solubility and preventing or dissolving protein aggregates [215,216,217].
Hydromethylthionine and methylene blue	Tau	Intermediate gel-like condensates	Promotes Tau LLPS and accelerates liquid-to-gel transitions while suppressing fibril formation and seeding [159,183,184,185].
Suramin	Tau	Tau:polyanion coacervate condensates	Modulates and reverses Tau coacervation with heparin/RNA and reduce seeding potential of aged coacervates [199].
Epigallocatechin gallate (EGCG)	Tau	Liquid and intermediate regimes	Modulates droplet material properties and reduces fibril formation or seeding [200].
EGCG	Aβ, Tau, α-Synuclein	Fibrillation (effect not yet shown specifically in condensate regimes)	Can bind monomeric proteins and reduce fibrillation or remodel existing fibrils [218,219,220,221,222].
Gallic acid	Tau	Liquid and intermediate regimes	Alters Tau condensate properties and reduces aggregation [166].
Gallic acid	Aβ, Tau, α-Synuclein	Fibrillation (effect not yet shown specifically in condensate regimes)	Binds to monomer- and oligomeric proteins and prevents fibrillation [223,224,225].
Tannic acid	Tau	Liquid and intermediate regimes	Promotes or suppresses LLPS in a concentration-dependent manner and alters condensate liquidity [165].
Tannic acid	Aβ, α-Synuclein	Fibrillation (effect not yet shown specifically in condensate regimes)	Prevents amyloid fibrillation pathways [226,227].
Claramine	α-Synuclein	Cytoplasmic liquid condensates	Stabilizes condensate state while suppressing amyloid conversion within droplets [38].
Curcumin	α-Synuclein	Pathological condensates	Suppresses fibrillization/maturation within condensates [201].
Curcumin	Aβ, Tau, α-Synuclein	Fibrillation (not yet shown specifically in condensate regimes)	Suppresses fibrillization pathways and binds to existing fibrils [228,229,230,231].

## 5. Conclusions

Here, we highlight that amyloid formation is not dictated by sequence alone, but by the molecular and physicochemical context in which that sequence is engaged. In fact, the same regions that support physiological condensation, molecular organization, and functional partner recruitment can, under shifts in stoichiometry, mutation, stress, aging, or interfacial interactions, be rerouted toward β-rich oligomers and fibrillar assemblies. By considering the ND-related proteins Tau, α-synuclein, Aβ, TDP-43, FUS, and hnRNPA1 together, we emphasize that the relationship between LLPS and amyloid is not inherently protective or inherently pathological, but strongly dependent on context, composition, and material state. These perspectives explain how condensates can support normal biology while also creating conditions that favor toxic self-assembly. Moving forward, a major challenge will be not only to define which condensate states are functional and which are truly aggregation-prone, but also to determine how pathological condensates can be targeted selectively without disrupting the normal assemblies required for cellular function. Addressing that problem will require more precise structural, temporal, and mechanistic resolution of condensate maturation and may ultimately reshape how we think about therapeutic intervention in protein misfolding disease.

## Data Availability

No data was generated in the preparation of this review article.
